# Loss of cytoplasmic survivin expression is an independent predictor of poor prognosis in radically operated prostate cancer patients

**DOI:** 10.1002/cam4.2773

**Published:** 2020-01-01

**Authors:** Franziska Büscheck, Mariam Sulimankhil, Nathaniel Melling, Doris Höflmayer, Claudia Hube‐Magg, Ronald Simon, Cosima Göbel, Andrea Hinsch, Sören Weidemann, Jacob R. Izbicki, Frank Jacobsen, Tim Mandelkow, Niclas C. Blessin, Christina Möller‐Koop, Florian Lutz, Florian Viehweger, Katharina Möller, Guido Sauter, Maximillian Lennartz, Eike Burandt, Patrick Lebok, Sarah Minner, Sarah Bonk, Hartwig Huland, Markus Graefen, Thorsten Schlomm, Christoph Fraune

**Affiliations:** ^1^ Institute of Pathology University Medical Center Hamburg‐Eppendorf Hamburg Germany; ^2^ General, Visceral and Thoracic Surgery Department and Clinic University Medical Center Hamburg‐Eppendorf Hamburg Germany; ^3^ Martini‐Clinic Prostate Cancer Center University Medical Center Hamburg‐Eppendorf Hamburg Germany; ^4^ Department of Urology Charité–University Medical Center Berlin Berlin Germany

**Keywords:** BIRC5, deletion, ERG, prostate cancer, Survivin, TMA

## Abstract

Survivin is an inhibitor of apoptosis. Aberrant survivin expression occurs in malignant tumors and has often been linked to unfavorable patient outcome. Here we analyzed 12 432 prostate cancers by immunohistochemistry. Survivin immunostaining was regularly expressed at high levels in normal prostate epithelium but expression was often reduced in prostate cancers. Among 9492 evaluable prostate cancers, 9% expressed survivin strongly, 19% moderately, 28% weakly, and 44% lacked it. Loss of cytoplasmic survivin was seen in advanced tumor stage, higher Gleason score, preoperative PSA levels, and Ki‐67 labeling index, and associated with earlier PSA recurrence (*P* < .0001). Survivin loss was significantly more common in cancers carrying *TMPRSS2:ERG* fusions (61% survivin negative) than in ERG wild‐type cancers (32% survivin negative; *P* < .0001). Multivariate analysis revealed that reduced cytoplasmic survivin expression predicted poor prognosis independent from Gleason score, pT, pN, and serum PSA level. This was valid for ERG‐positive and ERG‐negative cancers. Survivin expression loss even retained its prognostic impact in 1020 PTEN deleted cancers, a group that is already characterized by dismal patient prognosis. In conclusion, reduced survivin expression is associated with more aggressive tumors and inferior prognosis in prostate cancer.

## Graphical abstract

Survivin encoded by the BIRC5 gene was analyzed by immunochemistry on a tissue microarray of 12 432 prostate cancers. We found loss of survivin expression linked to early PSA recurrence in patients treated with radical prostatectomy. Loss of survivin expression showed a moderate and independent impact on PSA recurrence‐free survival. This effect remained also in PTEN‐deleted cancers.

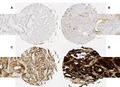

## 1 INTRODUCTION

Prostate cancer (PCa) is the most frequently diagnosed male cancer.^1^ While many PCa are characterized by a favorable behavior, some are highly aggressive.^2^, ^3^ Established prognostic criteria are Gleason grade and tumor extent at biopsy, increasing prostate‐specific antigen (PSA) level, and clinical stage. Although statistically powerful, they are suboptimal for individual treatment choices. Thus, it is hoped that additional molecular markers will enable a more precise prediction of PCa aggressiveness.

The 16.5 kDa survivin molecule is encoded by the *BIRC5* gene. Its functional role includes inhibition of apoptosis in concert with promotion of cell proliferation as a central regulator of spindle formation and enhancement of tumor angiogenesis.^4^, ^5^ The exact mechanism by which survivin exerts these functions are not fully understood. Survivin typically exists in the two distinct subcellular pools of the cytoplasm and the nucleus.^6^ Survivin expression has predominantly been reported in fetal tissues, such as intestinum, liver, kidney, epidermis, spleen, thymus and placenta.^7^ Several adult normal cells also express survivin, such as basal colonic epithelial cells, thymocytes, and bone marrow‐derived stem cells.^8^ Survivin expression has also been found in a wide variety of malignomas, including breast cancer, colorectal cancer, bladder cancer, and lung cancer.^7^, ^9^, ^10^ In most of these tumor entities, elevated expression has been linked with biologically aggressive cancer subtypes and poor prognosis.^4^, ^8^, ^11^ In the prostate, survivin expression has been reported in nonmalignant neuroendocrine cells,^12^ cancer cell lines,^13^ and androgen‐dependent as well as androgen‐independent cancer tissues.^14^, ^15^ Multiple studies on PCa showed that survivin is upregulated in PCa as compared to normal prostate epithelium. Whether high levels of survivin expression are linked to aggressive tumor phenotype and poor patient prognosis is debated.^11^, ^14^, ^15^, ^16^, ^17^, ^18^, ^19^, ^20^, ^21^

To further clarify the clinical significance of survivin expression, a preexisting PCa tissue microarray (TMA)^22^, ^23^, ^24^ was analyzed in this study. The results identify a moderate prognostic role of survivin expression, which is independent of established clinical and pathological parameters.

## 2 MATERIAL AND METHODS

### 2.1 Patients

Radical prostatectomy (RPE) specimens were available from 12 427 patients with surgery between 1992 and 2012 (Department of Urology and the Martini Clinic at the University Medical Center Hamburg‐Eppendorf). All specimens were analyzed according to a standard procedure.^23^ Follow‐up was available for 11 152 patients (median 60 months, range 1 to 241 months; Table 1). After RPE, prostate‐specific antigen (PSA) level was measured regularly. PSA recurrence was defined as the time point when PSA reached 0.2 ng/mL. The TMA manufacturing process was described earlier in detail.^25^ In short, one 0.6 mm core was taken from a representative tissue block from each patient. For internal controls, the TMA contained various control tissues, including normal prostate. The TMA was annotated with results on ERG expression, *ERG* break apart FISH analysis^26^ and deletion status of 10q23 (*PTEN*),^27^ 6q15 (*MAP3K7*),^28^ 5q21 (*CHD1*),^28^ 3p13 (*FOXP1*),^29^ and Ki‐67 labeling index (Ki‐67 LI).^30^ Archived diagnostic leftover tissues was used together with anonymized data, approved by the local ethics committee (Ethics commission Hamburg, WF‐049/09) and in accordance with the local laws (HmbKHG, §12a). All work has been carried out in compliance with the Helsinki Declaration.

**Table 1:** Pathological and clinical data of the arrayed prostate cancers

	No. of patients (%)	
	Study cohort on TMA*	Biochemical relapse
Follow‐up (month)		
N	11 152	2769 (24.8%)
Mean/median	64.4/60.0	—
Age (y)		
≤50	323	81 (25.1%)
51‐59	2696	705 (26.1%)
60‐69	6528	1610 (24.7%)
≥70	1498	370 (24.7%)
Pretreatment PSA (ng/mL)		
<4	1585	242 (15.3%)
4‐10	7480	1355 (18.1%)
10‐20	2412	737 (30.6%)
>20	812	397 (48.9%)
pT stage (AJCC 2002)		
pT2	8187	1095 (13.4%)
pT3a	2660	817 (30.7%)
pT3b	1465	796 (54.3%)
pT4	63	51 (81.0%)
Gleason grade		
≤3 + 3	2297	230 (10.0%)
3 + 4	6679	1240 (18.6%)
3 + 4 Tertiary 5	433	115 (26.6%)
4 + 3	1210	576 (47.6%)
4 + 3 Tertiary 5	646	317 (49.1%)
≥4 + 4	416	348 (83.7%)
pN stage		
pN0	6970	1636 (23.5%)
pN+	693	393 (56.7%)
Surgical margin		
Negative	9990	1848 (18.5%)
Positive	2211	853 (38.6%)

Abbreviation: AJCC, American Joint Committee on Cancer.

* Numbers do not always add up to 12 432 in different categories because of cases with missing data.

### 2.2 Immunohistochemistry

**Figure 1:**
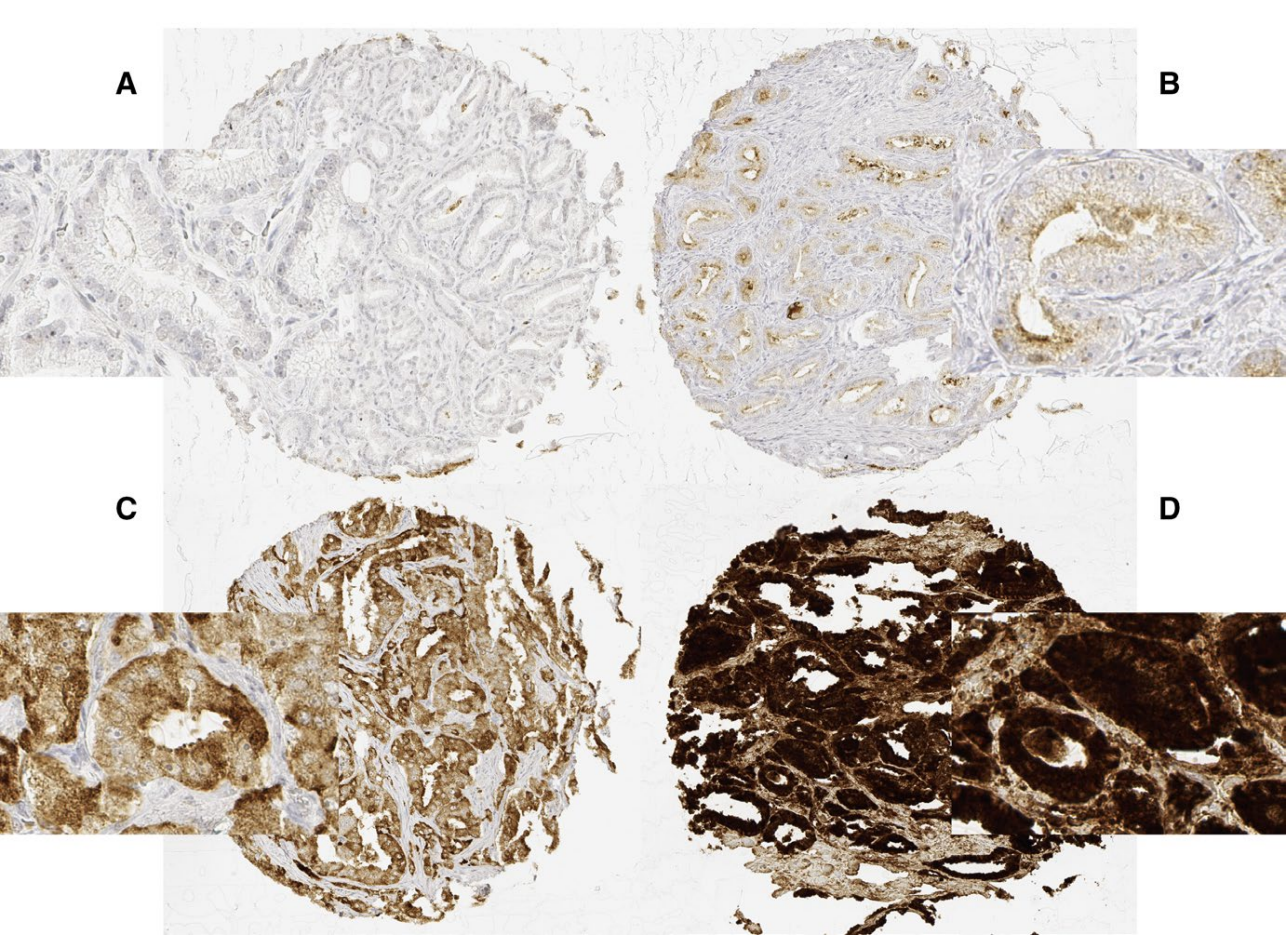
Representative pictures of survivin immunostaining in prostate cancer with (A) negative, (B) weak, (C) moderate, and (D) strong staining. Magnification 100×/400×, TMA spot size 600 μm

Freshly cut TMA sections were analyzed in one experiment on a single day. TMA sections were deparaffinized followed by heat treatment (5 minutes, 120°C, pH 7.8 Tris‐EDTA‐citrate buffer). Primary rabbit monoclonal anti‐survivin antibody EP2880Y (Abcam ab76424) was used in a final dilution of 1:900. Survivin staining was visualized utilizing the Envision System (DAKO, Glostrup). Survivin staining was recorded as described^26^: Negative scores showed no staining at all. Weak was defined as a staining intensity of 1+ in ≤70% of the tumor cells or a staining intensity of 2+ in ≤30% of the tumor cells; moderate as a staining intensity of 1+ in>70% of tumor cells, a staining intensity of 2+ in >30% but in ≤70% of the tumor cells or a staining intensity of 3+ in ≤30% of the tumor cells; and strong as a staining intensity of 2+ in >70% of the tumor cells or a staining intensity of 3+ in >30% of the tumor cells (Figure 1). While the anti‐survivin monoclonal antibody EP2880Y and EP119 recognized in bladder cancer nuclear survivin, staining in prostate cancer was mainly cytoplasmic (Figure S1).

### 2.3 Statistics

Pearson's chi‐square test for contingency tables was calculated. Kaplan‐Meier survival curves were evaluated with the log rank test. Multivariate analysis was accomplished utilizing the Cox regression model to identify independence of accepted clinical parameters and survivin expression pattern as well as expression intensities to predict PSA recurrence‐free survival. We used JMP 12 (SAS Institute Inc).

## 3 RESULTS

### 3.1 Technical issues

76% of tumor samples was interpretable in the TMA analysis. Noninformative cases (2935; 24%) lacked the tissue sample or the TMA spot did not show an unequivocal cancer tissue.

### 3.2 Survivin expression in PCa

Normal prostate epithelium stained positive for survivin, predominantly localized in the cytoplasm. Occasionally staining presented an apical or a nuclear pattern. Survivin expression was lost in 4145 of 9492 (43.7%) PCa. Positive immunostaining was recognized in 5347 prostate carcinomas with 58.8% cytoplasmic, 35.6% apical, and 5.7% nuclear localization and considered strong in 16.1%, moderate in 33.8%, and weak in 50.0% of survivin‐positive cancers. Representative images of negative and positive survivin immunostainings are given in Figure 1.

### 3.3 Association with TMPRSS2:ERG fusion status and ERG protein expression

**Figure 2:**
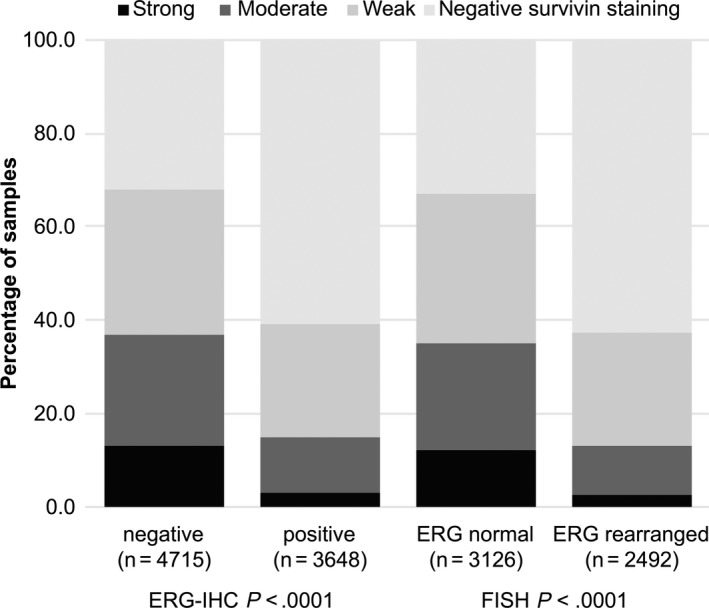
Association between survivin immunostaining and ERG‐status (IHC/FISH) in all cancers

Data on both ERG FISH and IHC were available from 5416 cancers, and showed a concordant result in 5170 of 5416 (95.5%) cancers. Absence of survivin staining was linked to *TMPRSS2:ERG* rearrangement and ERG expression in PCa. Survivin immunostaining was absent in 61% of cancers with *TMPRSS2:ERG* fusion detected by IHC and 63% of cancers detected by FISH, but only in 32% of cancers without ERG staining and 33% of cancers without ERG rearrangements detected by FISH (*P* < .0001 each; Figure 2).

### 3.4 Association with tumor phenotype

Loss of survivin was associated with higher Gleason score, advanced pT stage, lymph node positivity, and higher preoperative PSA level and positive resection margin status. These associations also hold true in the subset of *ERG*‐negative and *ERG*‐positive cancers (*P* < .0001 each; Table 2 and Tables S1 and S2).

**Table 2:** Association between survivin staining results and prostate cancer phenotype in all cancers

Parameter	N	Survivin (%)				*P*
		Negative	Weak	Moderate	Strong	
All cancers	9492	43.7	28.2	19.1	9.1	
Tumor stage						<.0001
pT2	6114	35.1	31.4	22.4	11.1	
pT3a	2130	54.2	24.7	14.6	6.5	
pT3b‐pT4	1213	67.8	18.2	10.0	4.0	
Gleason grade						<.0001
≤3 + 3	2102	29.0	31.7	26.9	12.4	
3 + 4	5398	42.4	30.1	18.6	8.9	
4 + 3	1499	59.8	20.6	12.7	6.9	
≥4 + 4	444	73.4	13.1	9.7	3.8	
Lymph node metastasis						<.0001
N0	5308	47.2	28.1	16.8	7.9	
N+	550	73.8	16.2	6.4	3.6	
Preoperative PSA level (ng/mL)						<.0001
<4	1152	39.2	29.4	22.1	9.2	
4‐10	5680	40.7	29.7	19.9	9.6	
10‐20	1903	49.6	25.4	16.5	8.5	
>20	664	58.1	21.7	14.2	6.0	
Surgical margin						<.0001
Negative	7531	41.1	29.4	19.9	9.5	
Positive	1787	53.4	22.8	16.2	7.6	

### 3.5 Association with genomic deletions

**Figure 3:**
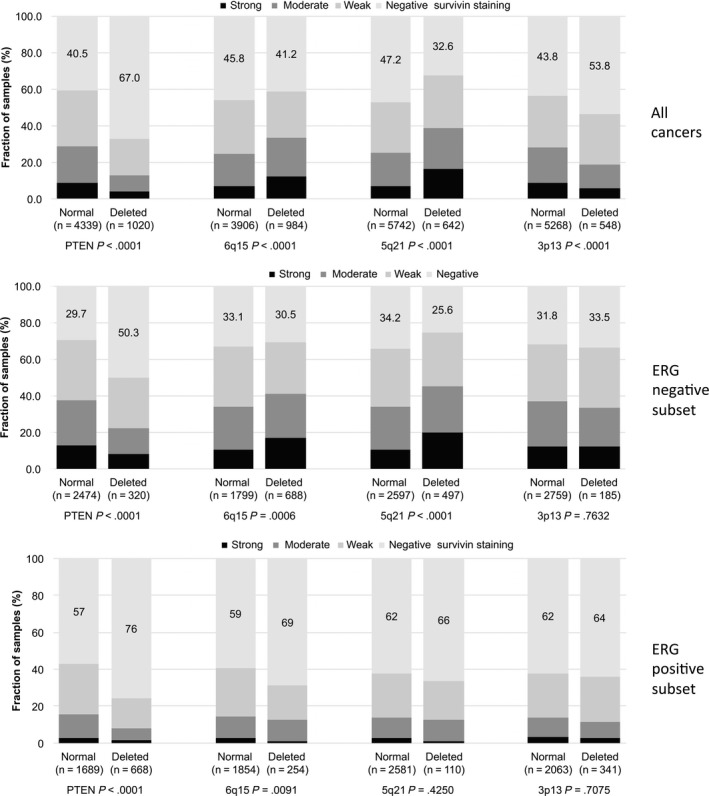
Association between surviving expression and 10q23 (*PTEN*), 5q21 (*CHD1*), 6q15 (*MAP3K7*), 3p13 (*FOXP1*) deletion in all cancers, the ERG‐positive and the ERG‐negative subset

To study, whether survivin expression might be associated with one or several genomic deletions, survivin data were compared with 10q23 (*PTEN*), 3p13, 6q15, and 5q21 deletion data. This analysis revealed several significant associations in the combined analysis of all cancers but also in the subsets of *ERG*‐positive and ERG‐negative cancers (Figure 3).

### 3.6 Association with Ki‐67 labeling index

Reduced or absent survivin staining was significantly associated with cell proliferation as measured by Ki‐67 LI. This was also seen in the subsets of Gleason score ≤3 + 3 and 3 + 4 (*P* < .0001 each; Table 3) cancers but not in Gleason grade 4 + 3 and ≥ 4 + 4 cancers.

**Table 3:** Association between survivin staining and Ki‐67 labeling index in Gleason groups

Gleason	Survivin	N	Ki‐67 LI	(Mean ± SEM)	*P*
Total	Negative	2547	3.04	0.05	<.0001
	Weak	1610	2.60	0.07	
	Moderate	1077	2.47	0.08	
	Strong	513	2.14	0.12	
≤3 + 3	Negative	381	2.59	0.10	<.0001
	Weak	393	2.10	0.10	
	Moderate	340	2.06	0.11	
	Strong	153	1.44	0.16	
3 + 4	Negative	1479	2.90	0.06	<.0001
	Weak	996	2.59	0.07	
	Moderate	593	2.38	0.10	
	Strong	286	2.07	0.14	
4 + 3	Negative	502	3.42	0.16	=.5040
	Weak	180	3.39	0.26	
	Moderate	113	3.48	0.33	
	Strong	61	4.13	0.45	
≥4 + 4	Negative	170	4.26	0.34	=.4451
	Weak	31	4.58	0.79	
	Moderate	26	5.35	0.86	
	Strong	11	2.91	1.32	

### 3.7 Association with PSA recurrence

**Figure 4:**
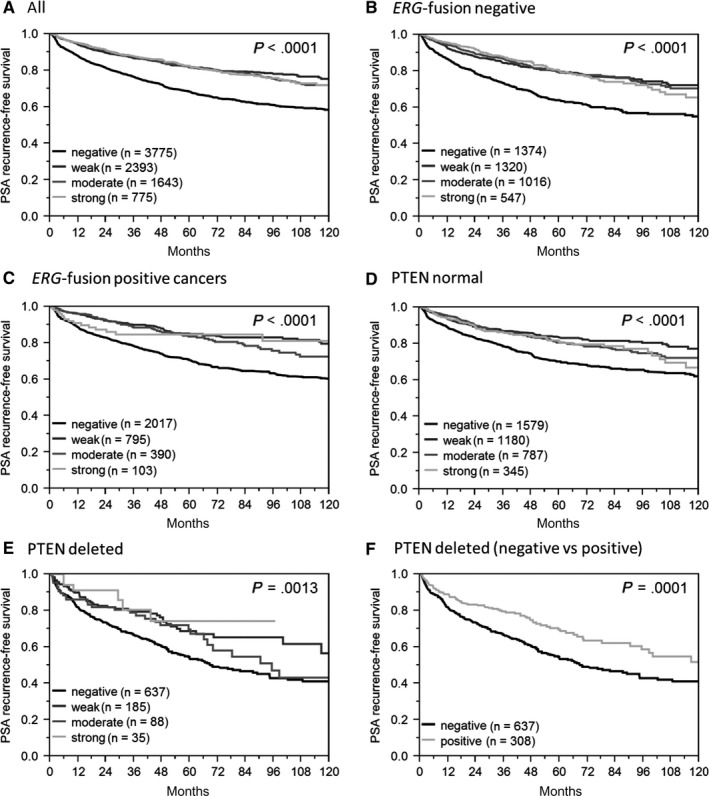
Association between survivin *expression* and biochemical recurrence in (A) all cancers, (B) *ERG*‐fusion negative cancers, (C) *ERG*‐fusion positive cancers, (D) *PTEN* normal cancers, (E) *PTEN*‐deleted cancers, (F) *PTEN*‐deleted cancers (negative vs positive)

Reduced survivin expression was associated with early PSA recurrence (*P* < .0001; Figure 4A). This also applied to the subset of *ERG*‐negative and *ERG*‐positive cancers (*P* < .0001 each; Figure 4B,C) as well as in cancers without (*P* < .0001; Figure 4D) and with PTEN deletion (*P* = .0013; Figure 4E).

### 3.8 Multivariate analysis

Cox regression analysis was done in four scenarios (Table 4). Model 1 and 2 evaluated postoperatively available parameters. Model 3 was a mixture of pre and postoperative parameters. Model 4 included the preoperative Gleason score obtained on the original biopsy combined with preoperative PSA, cT stage, and survivin expression. These analyses showed, that survivin was independent prognosticator in all preoperative scenarios (*P* < .05). The univariate Cox proportional hazard ratio for PSA recurrence‐free survival of negative vs strong survivin staining was a moderate 2.03 (Table S3).

**Table 4:** Multivariate analysis with established prognostic parameters and the surviving expression in all cancers, the ERG‐negative and ‐positive subset

Subset	Scenario	N	Log‐rank *P*							
			Preoperative PSA‐Level	pT stage	cT stage	Gleason grade prostatectomy	Gleason grade biopsy	pN stage	R status	Survivin expression
All cancers	1	5158	<.0001	<.0001	—	<.0001	—	<.0001	.0080	.1494
	2	8385	<.0001	<.0001	—	<.0001	—	—	<.0001	.0343
	3	8283	<.0001	—	<.0001	<.0001	—	—	—	<.0001
	4	8172	<.0001	—	<.0001	—	<.0001	—	—	<.0001
ERG‐negative	1	2630	<.0001	<.0001	—	<.0001	—	.0021	.3001	.3905
	2	4160	<.0001	<.0001	—	<.0001	—	—	.0016	.3119
	3	4133	<.0001	—	<.0001	<.0001	—	—	—	.0123
	4	4078	<.0001	—	<.0001	—	<.0001	—	—	.0002
ERG‐positive	1	2014	.0012	<.0001	—	<.0001	—	.0467	.0144	.0491
	2	3229	<.0001	<.0001	—	<.0001	—	—	<.0001	.0925
	3	3165	<.0001	—	<.0001	<.0001	—	—	—	.0045
	4	3126	<.0001	—	<.0001	—	<.0001	—	—	<.0001

Note

Scenario 1 includes all postoperatively available parameters (pathological tumor (pT) stage, lymph node status (pN), surgical margin (R) status, preoperative PSA value, and Gleason grade obtained after the morphological evaluation of the entire resected prostate. Scenario 2 excluded the nodal status from analysis. Scenario 3 included preoperative PSA, clinical tumor (cT) stage and Gleason grade obtained on the prostatectomy specimen. In scenario 4, the preoperative Gleason grade obtained on the original biopsy was combined with preoperative PSA, and cT stage.

## 4 DISCUSSION

This study shows, that reduced survivin expression is associated with less favorable outcome after prostatectomy.

The available data from the literature have demonstrated, that the biological and clinical role of survivin expression varies depending on the tumor type. Associations between high survivin levels and poor prognosis have been described in esophageal, pancreatic, and bladder cancer.^31^, ^32^, ^33^ In breast and gastric cancer, reduced survivin levels were linked to unfavorable outcome.^34^, ^35^ In this study, immunohistochemical survivin staining was regularly seen in normal prostate epithelium but was lost in 44% of 9492 interpretable PCa in our study. Considering also significant associations between reduced survivin expression and unfavorable tumor features such as high Gleason grade, advanced pT stage, or lymph node metastasis, these findings argue for a role of survivin expression loss in PCa development and progression.

Several lines of evidence support the validity of our data. The antibody (rabbit monoclonal anti‐survivin, Epitomics clone EP2880Y, Abcam ab76424) utilized in this study has been thoroughly tested for specificity by Western blot, flow cytometry, immunoprecipitation, and sandwich ELISA according to the product datasheet. In other tumor types, the same antibody resulted in associations between high survivin levels and poor prognosis, such as in studies on ovarian cancers^36^ and papillary thyroid cancers^37^, ^38^ This is comparable to what has been found using other anti‐survivin antibodies. For example, a link of high survivin levels to poor outcome in ovarian cancers was also found using anti‐survivin EP119,^39^ and associations between survivin overexpression and high grade, advanced stage and metastasis of thyroid cancers was reported with another rabbit anti‐survivin from Boster Biological Technology^40^ It is of note, that several other studies analyzing 82 to 114 PCa suggested that the cytoplasmic expression of survivin might represent an indicator for bad prognosis in this tumor type (Table S4).^16^, ^17^, ^20^, ^21^, ^41^ One more recent analysis that also included some of the tumors of our patient cohort described a marginal link of “altered” survivin expression with unfavorable patient prognosis by utilizing an early digital image analysis system.^41^ However, this study differed from the present one using a polyclonal anti‐survivin antibody and instead of loss of cytoplasmic expression of survivin it used “altered” expression of survivin as readout (>10% of cells were cytoplasmic or nuclear stained).

Our PCa cohort has been analyzed extensively in the past, which resulted in a considerable quantity of molecular data available for further analysis. For the purpose of this study, we were interested in the relationship of survivin expression with *TMPRSS2:ERG* gene fusion, which is the most common molecular event in PCa and PTEN deletions one of the strongest prognostic features in this tumor, as well as tumor cell proliferation measured by the Ki‐67 labeling index. TMPRSS2:ERG fusions occur in about 50% of PCa, predominantly in younger patients, and lead to a constituitive overexpression of the transcription factor ERG.^42^ ERG overexpression by itself lacks prognostic relevance,^26^ but modulates the expression of more than 1600 genes in prostate epithelial cells. Our data suggest that loss of survivin is ERG dependent as survivin protein levels were clearly lower in ERG‐positive than in ERG‐negative cancers. The lower levels of survivin expression in PTEN‐deleted cancers are consistent with a functional interaction between survivin and the PTEN/AKT pathway. That the interaction with PTEN is not responsible for the prognostic impact of reduced survivin expression is demonstrated by its retained prognostic role in PTEN deleted cancers. This is unusual and further argues for a particularly strong prognostic role of survivin expression loss in PCa. PTEN deletions are linked to poor prognosis in PCa.^27^ Many prognostic features fail to further stratify patient outcome in molecular subgroups that are already defined by PTEN deletion.^43^ The significant link between low‐level survivin staining and tumor proliferation would be consistent with a role of survivin expression in the control of cellular proliferation as suggested by several authors.^35^, ^44^ That association between survivin expression levels and Ki‐67 LI were not found in Gleason score 4 + 3 and ≥4 + 4 tumors might be caused by low case numbers in these particular groups.

The moderate independent prognostic role found for cytoplasmic survivin expression in this study suggests that measuring this protein could result in useful prognostic information for PCa patients. It is noteworthy, however, that prognostic parameters are needed for PCa patients that are not only independent of established factors but also better reproducible and thus more reliable. The Gleason grade, the most decisive preoperative prognosticator, suffers from interobserver variability reaching up to 40%. This also applies to expert pathologists.^45^

We consider it likely that the analysis of molecular markers will in the future complement the work‐up of prostate biopsies in patients for whom different therapeutic options exist. The advent of multiplex immunohistochemistry enabling the simultaneous application of multiple antibodies will make it possible to easily apply antibody cocktails to diagnostic biopsies. Our data suggest, that survivin protein quantitation may be part of such a future diagnostic system.

In summary, these data identify an association of reduced cytoplasmic survivin expression with unfavorable disease course in PCa. The statistical independence from all preoperatively and even postoperatively available prognostic parameters argues for a possible diagnostic application of survivin measurement.

## CONFLICT OF INTEREST

The authors declare no conflict of interest.

## Supporting information

Suppinfo

## Data Availability

The data that support the findings of this study are available from the corresponding author upon reasonable request.
