# Supplementary material for: Loss of cytoplasmic survivin expression is an independent predictor of poor prognosis in radically operated prostate cancer patients
Source: Cancer Med. 2020 Jan 1;9(4):1409–18. doi: 10.1002/cam4.2773 (PMC7013067; doi:10.1002/cam4.2773)
Supplement: Supplementary file 1 — Suppinfo [file CAM4-9-1409-s001.docx]

**Supplementary Table S1:** Association between survivin staining results and prostate cancer phenotype in ERG- negative cancers

| **Parameter** | **N** | **Survivin (%)** | | | | | **P** |  |
| --- | --- | --- | --- | --- | --- | --- | --- | --- |
|  |  | **Negative** | **Weak** | **Moderate** | **Strong** | |  |  |
| **All cancers** | 4715 | 31.9 | 31.2 | 23.9 | | 12.9 |  | |
|  |  |  |  |  | |  |  | |
| **Tumor stage** |  |  |  |  | |  | <0.0001 | |
| pT2 | 3166 | 24.6 | 33.4 | 26.9 | | 15.0 |  | |
| pT3a | 949 | 39.4 | 29.9 | 20.1 | | 10.5 |  | |
| pT3b-pT4 | 588 | 58.5 | 21.4 | 14.3 | | 5.8 |  | |
|  |  |  |  |  | |  |  | |
| **Gleason grade** |  |  |  |  | |  | <0.0001 | |
| ≤3+3 | 989 | 20.3 | 31.3 | 32.7 | | 15.7 |  | |
| 3+4 | 2641 | 28.4 | 34.1 | 23.9 | | 13.6 |  | |
| 4+3 | 800 | 45.4 | 27.0 | 17.5 | | 10.1 |  | |
| ≥4+4 | 267 | 68.5 | 15.0 | 11.6 | | 4.9 |  | |
|  |  |  |  |  | |  |  | |
| **Lymph node metastasis** |  |  |  |  | |  | <0.0001 | |
| N0 | 2715 | 34.2 | 31.9 | 22.4 | | 11.5 |  | |
| N+ | 265 | 66.0 | 20.4 | 7.9 | | 5.7 |  | |
|  |  |  |  |  | |  |  | |
| **Preoperative PSA level (ng/ml)** |  |  |  |  | |  | <0.0001 | |
| <4 | 489 | 28.8 | 32.5 | 24.9 | | 13.7 |  | |
| 4-10 | 2784 | 27.7 | 32.8 | 25.6 | | 13.9 |  | |
| 10-20 | 1032 | 38.7 | 28.7 | 21.1 | | 11.5 |  | |
| >20 | 374 | 48.4 | 25.9 | 17.6 | | 8.0 |  | |
|  |  |  |  |  | |  |  | |
| **Surgical margin** |  |  |  |  | |  | <0.0001 | |
| negative | 3747 | 29.3 | 32.4 | 25.0 | | 13.4 |  | |
| positive | 889 | 42.3 | 26.0 | 20.6 | | 11.1 |  | |

**Supplementary Table S2:** Association between survivin staining results and prostate cancer phenotype in ERG- positive cancers

| **Parameter** | **N** | **Survivin (%)** | | | | **P** |
| --- | --- | --- | --- | --- | --- | --- |
|  |  | **Negative** | **Weak** | **Moderate** | **Strong** |  |
| **All cancers** | 3648 | 60.9 | 24.3 | 11.6 | 3.2 |  |
|  |  |  |  |  |  |  |
| **Tumor stage** |  |  |  |  |  | <0.0001 |
| pT2 | 2152 | 52.5 | 28.8 | 14.5 | 4.3 |  |
| pT3a | 986 | 69.6 | 20.0 | 8.6 | 1.8 |  |
| pT3b-pT4 | 495 | 80.2 | 13.3 | 5.1 | 1.4 |  |
|  |  |  |  |  |  |  |
| **Gleason grade** |  |  |  |  |  | <0.0001 |
| ≤3+3 | 747 | 42.7 | 34.3 | 17.4 | 5.6 |  |
| 3+4 | 2185 | 60.8 | 25.0 | 11.3 | 2.9 |  |
| 4+3 | 561 | 79.5 | 12.3 | 6.6 | 1.6 |  |
| ≥4+4 | 134 | 85.1 | 7.5 | 6.0 | 1.5 |  |
|  |  |  |  |  |  |  |
| **Lymph node metastasis** |  |  |  |  |  | <0.0001 |
| N0 | 2059 | 66.0 | 22.8 | 9.1 | 2.1 |  |
| N+ | 227 | 82.8 | 10.6 | 4.8 | 1.8 |  |
|  |  |  |  |  |  |  |
| **Preoperative PSA level (ng/ml)** |  |  |  |  |  | <0.0001 |
| <4 | 497 | 52.3 | 27.2 | 16.3 | 4.2 |  |
| 4-10 | 2222 | 59.0 | 25.8 | 11.9 | 3.3 |  |
| 10-20 | 669 | 68.3 | 20.3 | 8.5 | 2.8 |  |
| >20 | 221 | 75.1 | 15.4 | 7.7 | 1.8 |  |
|  |  |  |  |  |  |  |
| **Surgical margin** |  |  |  |  |  | <0.0001 |
| negative | 2857 | 58.7 | 26.0 | 12.0 | 3.3 |  |
| positive | 723 | 68.7 | 18.0 | 10.4 | 2.9 |  |

**Supplementary** **Table S3:** Cox proportional hazards for PSA recurrence-free survival after prostatectomy of established preoperative prognostic parameter and survivin expression

| **Variable** |  | **N** | **Univariate analysis** | | **Multivariate analysis (N=8,445)** |
| --- | --- | --- | --- | --- | --- |
| **Gleason grade biopsy** | ≥4+4 vs. ≤3+3 | 12,172 | 5.91 (5.33-6.55) *** | 3.67 (3.22-4.18) *** | |
| **cT-stage** | T2c vs. T1c | 14,404 | 3.88 (3.25-4.59) *** | 2.04 (1.61-2.54) *** | |
| **Preoperative PSA-level** | ≥20 vs. <4 | 14,611 | 5.06 (4.41-5.81) *** | 3.53 (2.91-4.29) *** | |
| **Survivin expression** | Negative vs. strong | 8,728 | 2.03 (1.72-2.42) *** | 1.50 (1.27-1.80) *** | |
| **ERG negative subset** | Negative vs. strong | 4,335 | 2.09 (1.71-2.58) *** | - | |
| **ERG positive subset** | Negative vs. strong | 3,350 | 2.35 (1.49-4.03) *** | - | |

Confidence interval (95%) in brackets; asterisk indicate significance level: * p≤0.05, ** p≤0.001, *** p≤0.0001.

**Supplementary Table S4:** Overview on survivin studies in prostate cancer (Pca)

| Author | Year | Anti-survivin | Fraction of Pca | Localization | NPE | Localization | Prognosis HR>1 |
| --- | --- | --- | --- | --- | --- | --- | --- |
| Ambrosini | 1997 | Polyclonal | Yes |  |  |  |  |
| Krajewski | 2003 | Polyclonal | 78% | c | No |  | Low vs. high expression |
| Kaur | 2004 | Polyclonal | 82% | c |  |  | No |
| Shariat | 2004 | Polyclonal | 71% | c, (n) | 31% | c, (n) | Abnormal* vs. normal |
| Zhang | 2009 | Polyclonal | ~22% | n, c |  |  | Low vs. high expression |
| Rodriguez | 2010 | Monoclonal | Yes | c | No |  |  |
| Danilewicz | 2015 | Monoclonal | Yes | n, c |  |  |  |
| Eslami | 2016 |  | 99% | n, c | No |  |  |
| Mathieu | 2017 | Polyclonal |  |  |  |  | Altered* vs. normal |
| Büscheck | 2019 | Monoclonal | 56% | c, (n) | No/yes | c, (n) | Low vs. high expression |

NPE normal prostate epithelium; HR hazard ratio; c cytoplasmic, n nuclear staining

* Abnormal / altered means >10% of cells positive


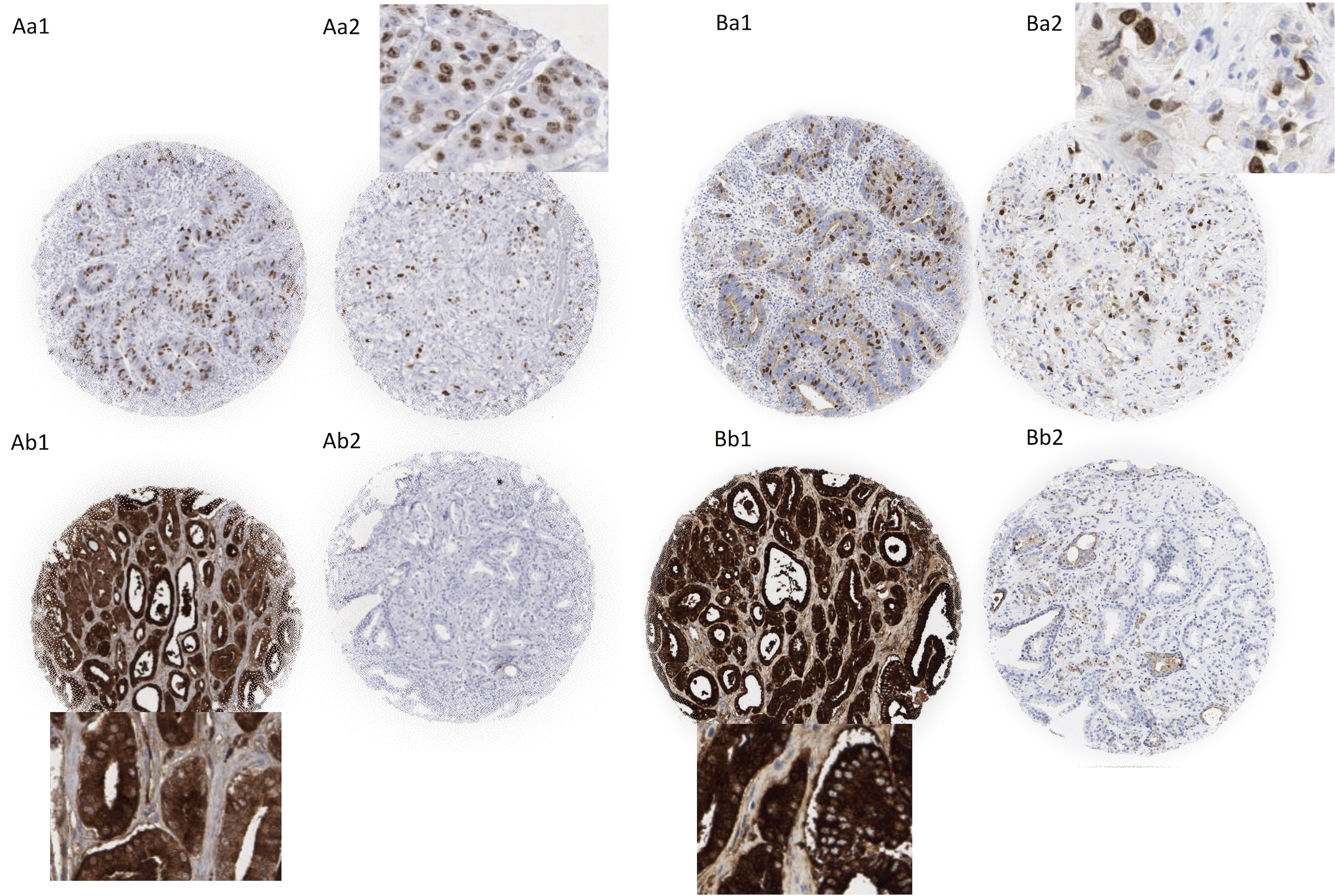


**Supplementary Fig. S1**: Anti-survivin staining with monoclonal antibody (A) EP2880Y and (B) EP 119 showed (a) nuclear staining in bladder cancer and (b) cytoplasmic staining in prostate cancer. (1) strong staining, (2) negative / weak staining.
